# Contextual Structured Reporting in Radiology: Implementation and Long-Term Evaluation in Improving the Communication of Critical Findings

**DOI:** 10.1007/s10916-020-01609-3

**Published:** 2020-07-28

**Authors:** Allard W. Olthof, Anne L. M. Leusveld, Jan Cees de Groot, Petra M. C. Callenbach, Peter M. A. van Ooijen

**Affiliations:** 1 Department of Radiology, Treant Health Care Group, Dr. G.H. Amshoffweg 1, Hoogeveen, The Netherlands; 2 Department of Radiation Oncology, University of Groningen, University Medical Center Groningen, Hanzeplein 1, Groningen, The Netherlands; 3 Department of Radiology, University of Groningen, University Medical Center Groningen, Hanzeplein 1, Groningen, The Netherlands; 4 Research Bureau, Treant Health Care Group, Dr. G.H. Amshoffweg 1, Hoogeveen, The Netherlands; 5 University Medical Center Groningen, Data Science Center in Health (DASH), Machine Learning Lab, University of Groningen, Zielstraweg 2, Groningen, The Netherlands

**Keywords:** Quality improvement, Radiology, Medical informatics, Communication

## Abstract

Structured reporting contributes to the completeness of radiology reports and improves quality. Both the content and the structure are essential for successful implementation of structured reporting. Contextual structured reporting is tailored to a specific scenario and can contain information retrieved from the context. Critical findings detected by imaging need urgent communication to the referring physician. According to guidelines, the occurrence of this communication should be documented in the radiology reports and should contain when, to whom and how was communicated. In free-text reporting, one or more of these required items might be omitted. We developed a contextual structured reporting template to ensure complete documentation of the communication of critical findings. The WHEN and HOW items were included automatically, and the insertion of the WHO-item was facilitated by the template. A pre- and post-implementation study demonstrated a substantial improvement in guideline adherence. The template usage improved in the long-term post-implementation study compared with the short-term results. The two most often occurring categories of critical findings are “infection / inflammation” and “oncology”, corresponding to the a large part of urgency level 2 (to be reported within 6 h) and level 3 (to be reported within 6 days), respectively. We conclude that contextual structured reporting is feasible for required elements in radiology reporting and for automated insertion of context-dependent data. Contextual structured reporting improves guideline adherence for communication of critical findings.

## Introduction

Structured reporting is a proven concept in radiology [1]. It contributes to an improved inter-reader agreement [2], improved communication [3], guideline compliance [4] and improved economic efficiency [5]. It facilitates data extraction [6], epidemiological research [7], and the development of deep learning algorithms [8]. Structured reporting is important in quality improvement programs, as indicated by 75% of respondents in an international survey [9].

Despite its broad application, different definitions for structured reporting are used interchangeably [10]. First, it can refer to standardized reporting where a template with well-established required components is used to make a structured report. This type of structured reporting improves quality by ensuring coherent and complete reports. The second definition of structured reporting refers to the way the content is arranged. Integrated into an IT-based workflow, this can contribute to improved usability and acceptance. Both components (content and structure) are needed to ensure that the structured reporting template contributes to quality improvement and contributes to efficient workflow. Suboptimal usability lowers adherence, and the potential quality improvement is not fully reached.

A drawback of structured reporting is the perceived inflexibility, limiting radiologists in using their creativity [11]. Radiologists can be forced to use a template that is not fully adapted to a specific situation. A solution for this is contextual structured reporting that is tailored to the clinical scenario [12]. In contextual structured reporting, different templates are used for various specific clinical scenarios. It can even refer to a more modular approach for different findings within clinical scenarios. With contextual structured reporting, a radiologist can use modular “building blocks” to customize a report to particular situations.

In this study, we worked out two concepts for context-dependent structured reporting, for both content and structure:

1. Structured reporting for a specific task within a radiology report using a sub-template
2. Automated insertion of data within this sub-template, dependent on contextual information

We report on the context-dependent structured reporting for the documentation of the communication of critical findings.

Critical findings are defined as findings in a radiological examination that need urgent patient management [13, 14]. Referring physicians need to be made aware of these findings as soon as possible. According to our national guideline, this communication should be documented in the radiology report and should contain three elements: a. by what method the communication took place, b. when the communication occurred, and c. to whom the findings were communicated. These items need to be included only in cases where critical findings are present and when this specific communication has taken place. In free-text reporting, there is the risk of omitting items. Structured reporting could help to increase adherence to guidelines [15], but its role in improving the communication of critical findings has not yet been investigated.

The purpose of this study is to:

1. develop a structured radiology report template with the automated insertion of context-dependent data elements.
2. implement this template in clinical practice to improve guideline adherence in the communication of critical findings.
3. evaluate the usability of this template by assessing long-term usage

## Methods

### Report template

**Fig. 1:**
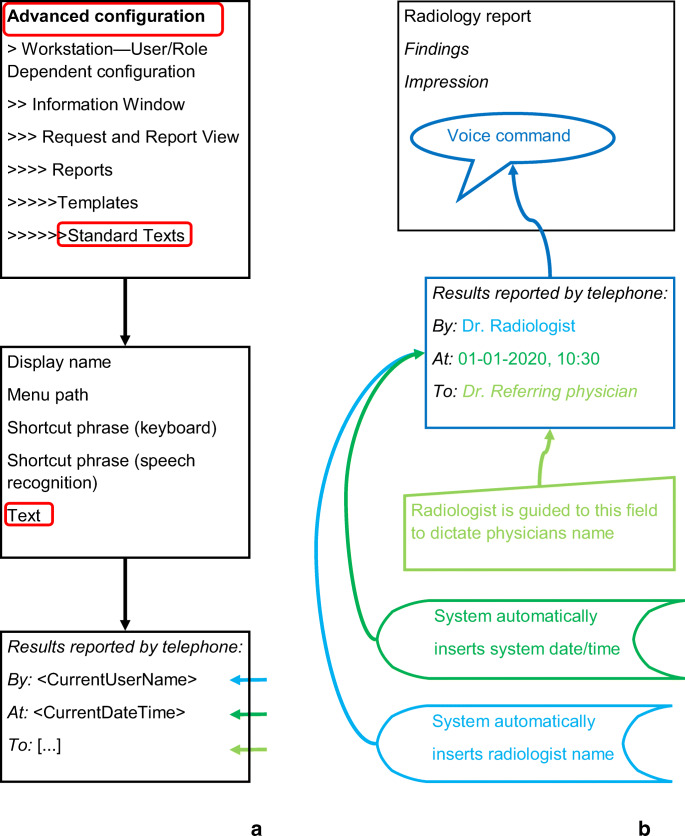
**a.** Schematic representation of the template-editor in the advanced configuration settings of the PACS (Sectra). Commands between <… > are executed when the template is activated; […] indicates a field were the radiologist dictates text. **b.** In his/her report the radiologist can insert the template by a voice command. The cursor is moved to the […]-field were the radiologist dictates the name of the referring physician. The “By:” and “At:” fields are automatically filled dependent on contextual information (radiologist username, and system data/time)

In June 2016, a local quality improvement project was started to facilitate radiologists in documenting their communication of critical findings in radiology reports according to our national guideline. For this purpose, an in-house structured reporting template was developed in the local PACS (IDS7 Sectra AB, Sweden) using the template editor of Sectra (Fig. 1a). The template was created by a local radiologist (AO) and made available to all radiologists of the three hospital locations of the Treant healthcare group.

The template consisted of three types of text: fixed, variable automated, and variable manual.

- Fixed text: text describing the method of communication, to ensure the ‘How’ -item of the guideline.
- Variable automated text dependent on the context:
  - code element that automatically inserts the actual date and time to ensure the ‘When’-item of the guideline.
  - code element that automatically inserts the name of the reporting radiologist.
- Variable manual data-element: name of the contacted physician, to ensure the ‘Who’-item of the guideline.

The template can be activated anywhere in a radiology report or in an addendum to ensure usability. To do so, the radiologist gives a voice-command to the reporting system (Fig. 1b) at the time of communication of critical findings to the referring physician. Radiologists had the choice to use this template or free-text to document this communication. The critical finding itself is not included in the template, but is described in the sections ‘findings’ and ‘impression’ of the report.

### Evaluation study

We performed a pre-implementation and post-implementation assessment. The collected data was restricted to reading process-related information not containing patient identifiers. Therefore, the local institutional review board waived the necessity of obtaining informed consent. The processing of the data was not “likely to result in a high risk”, according to the criteria in the Guidelines on Data Protection Impact Assessment (DPIA). Therefore a DPIA was not required [16].

We collected and compared data from three different time periods: July 2014 – June 2015 (before the publication of the national guideline), July 2016 – June 2017 (short-term post-implementation) and January 2020–13 March 2020 (long-term post-implementation).

Radiology reports were retrieved from the PACS to Excel by a text-mining query on Dutch words related to the communication of critical findings. The translated words/phrases are ‘by phone’, ‘called’, ‘verbally’, ‘passed on’, ‘telephone’, ‘discussed’, and ‘email’.

**Fig. 2:**
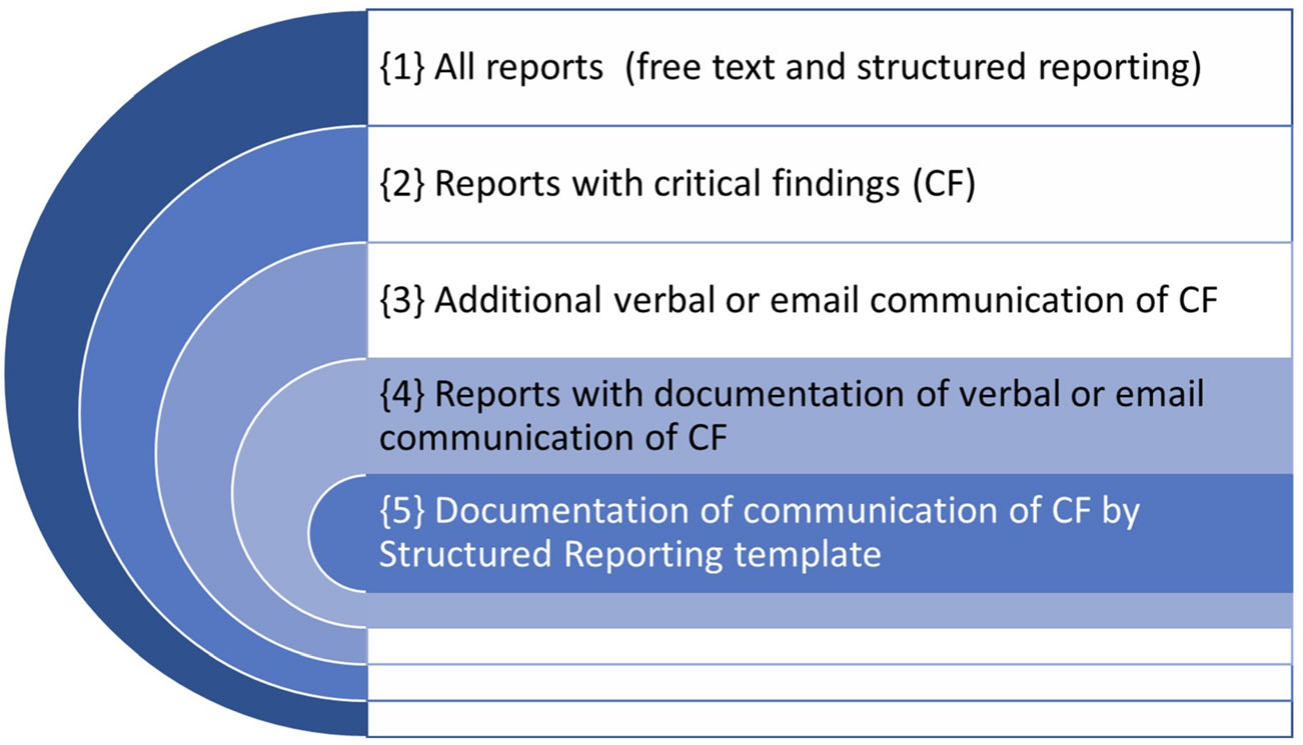
Documentation of critical findings in radiology reports. Radiologist are required to provide referring physicians an additional verbal or email notification of critical findings {3}, next to the written report {2}. This communication should, but is not always, documented in the radiology report {4} according to guidelines. The introduction of a small structured reporting template {5} facilitates the completeness of this documentation, and can be inserted by a voice command, regardless the form of the rest of the report (free-text or structured reporting). The number of reports in {1}, {4}, and {5} are assessed in the current study

The total number of reports per time period were recorded to calculate the percentage of reports with documented communication of critical findings. Reports with undocumented communication, or follow-up on critical findings without documented communication were beyond the scope of our study. Figure 2 indicates the hierarchical subgroups ranging from all reports to reports with documented critical findings using the structured reporting template.

The retrieved radiology reports were manually checked, and reports containing critical findings were included. A radiologist (AO) scored the included reports for the presence of the three required items according to the guideline:

- WHO: the name or role of the person to whom was communicated.
- WHEN: the date and time of the communication.
- HOW: the method of communication (direct verbal communication, telephone, or e-mail). These options for communication are based on the institutional working agreement. In case of e-mail communication, an acknowledgment of receipt should be recorded in the radiology report as well.

**Fig. 3:**
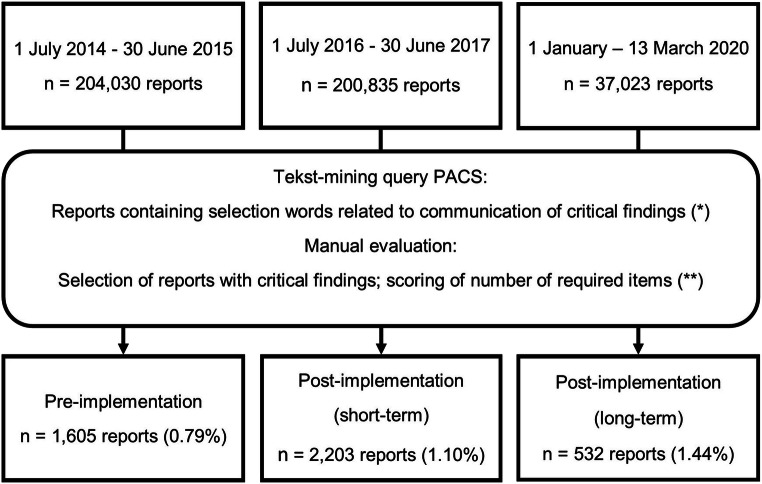
Flowchart of data-collection and processing. (*) The translated words\phrases are *by phone*, *called*, *verbally*, *passed on*, *telephone*, *discussed*, and *email*. (**) The required items are a description of who was communicated with (WHO), the date and time (WHEN), and the method of communication (HOW)

We scored whether or not the template was utilized. The flowchart of data collection is provided in Fig. 3.

The overall completeness of the information provided in the reports was assessed by adding the scored items per report ranging from 0 (none of the required items described), to 3 (all three items described). The results per radiologist were calculated by averaging the summed scores per time-period.

The content of the radiology reports was analysed by scoring the type of critical finding and the urgency level. According to the national guideline, level 1 represents life threatening critical findings that need to be communicated without delay within 60 min. Level 2 findings are to be communicated within 6 h, and level 3 findings within 6 days.

Statistical analyses were performed using SPSS. The presence of the items WHO, WHEN, and HOW in radiology reports pre-implementation were compared with short-term and long-term post-implementation using the Chi-Square Test. A *p* < 0.05 was considered to be statistically significant.

## Results

The proportion of radiology reports containing communication of critical findings increased after the implementation of a structured reporting template from 0.79% in the pre-implementation period to respectively 1.10% and 1.44% in the short-term and long-term post-implementation periods (Fig. 3).

**Fig. 4:**
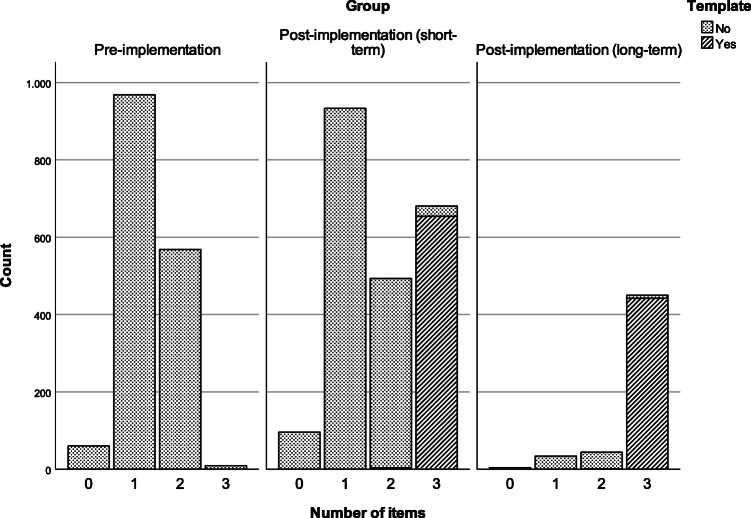
Absolute number of reports in three time periods with 0, 1, 2 or 3 of the required report items. In both post-implementation time periods usage of the structured reporting template is indicated. The length of the post-implementation (long-term) period was 73 days, both other periods 1 year

The reporting template usage increased from 30% of reports in the short-term post-implementation group to 83% of reports in the long-term post-implementation group (Table 2). Reports containing all three elements (WHO, WHEN, and HOW) were nearly absent in the pre-implementation period and increased by template usage in the short-term and long-term post-implementation periods (Fig. 4). When the template was used, the completeness of the description of the communication of critical findings increased significantly in the short-term and long-term post-implementation period for all three items. The highest increase was seen for the WHEN-item (from 2.5% to 100%, Table 1).). The WHO-item was present in 99.7% of cases when the template was used short-term post-implementation, despite requiring input from the radiologist. This increased to 100% long-term post-implementation. The HOW-item was around 50% in all groups without and around 100% in all groups with the template.

**Table 1:** Presence of the items WHO, WHEN, and HOW in radiology reports containing communication of critical findings pre-implementation compared with short-term and long-term post-implementation

	Pre-implementation	Post-implementation(short-term)			Post-implementation(long-term)			
	Without template (I)	Without template (II)	With template (III)		With template (IV)	With template (V)		
	*N* = 1605(%)	*N* = 1546(%)	*N* = 657(%)	*P* value (*)	*N* = 89(%)	*N* = 443(%)	P value (*)	
WHO	1332(83.0)	1152(74.5)	655(99.7)	<0.001	80(89.9)	443(100)	<0.001	
				I vs II: <0.001			I vs IV: 0.089	II vs IV0.001
				I vs III: <0.001			I vs V < 0.001	III vs V: 0.25
				II vs III: <0.001			IV vs V < 0.001	
WHEN	40(2.5)	62(4.0)	656(99.8)	<0.001	19(21.3)	443(100)	<0.001	
				I vs II: 0.02			I vs IV: <0.001	II vs IV: <0.001
				I vs III: <0.001			I vs V: <0.001	III vs V: 0.41
				II vs III: <0.001			IV vs V < 0.001	
HOW	759(47.3)	780(50.5)	657(100)	<0.001	45(50.6)	442(99.8)	<0.001	
				I vs II: 0.08			I vs IV: 0.55	II vs IV: 0.98
				I vs III: <0.001			I vs V: <0.001	III vs V: 0.22
				II vs III: <0.001			IV vs V: <0.001	

*= Chi-Square Test

A visual impression of the changes between the study periods is presented in Table 2 by color-coding the average number of items and the usage percentages of the template ranging from low (red) to high (green).

**Table 2.**
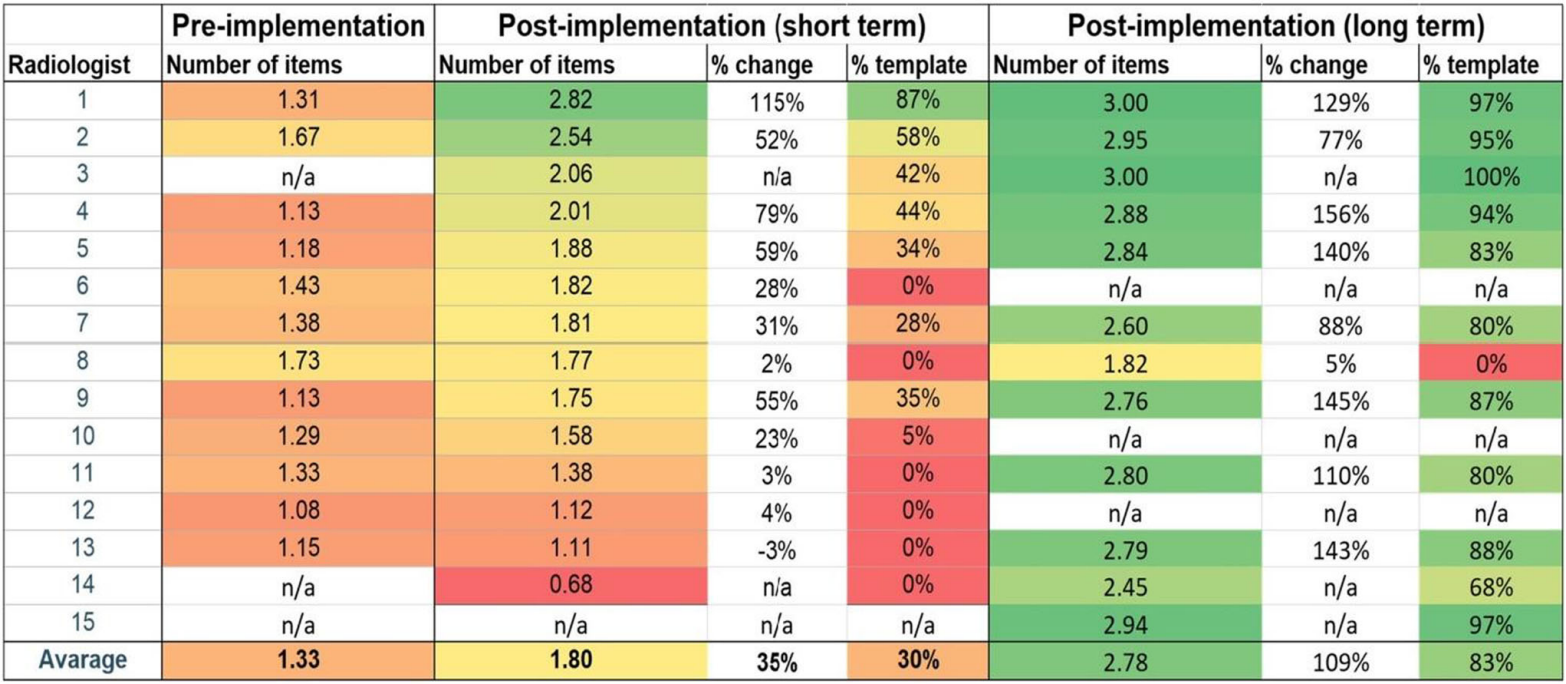
Presence of the total number of items (WHO, WHEN, and HOW) in radiology reports containing communication of critical findings.

n/a = radiologist did not work in the research institution that particular time period. % change post-implementation is calculated compared with pre-implementation. % template is the percentage of reports were the template was used. The color-coding from red to green indicates an increasing average number of items, and an increasing percentage

**Fig. 5:**
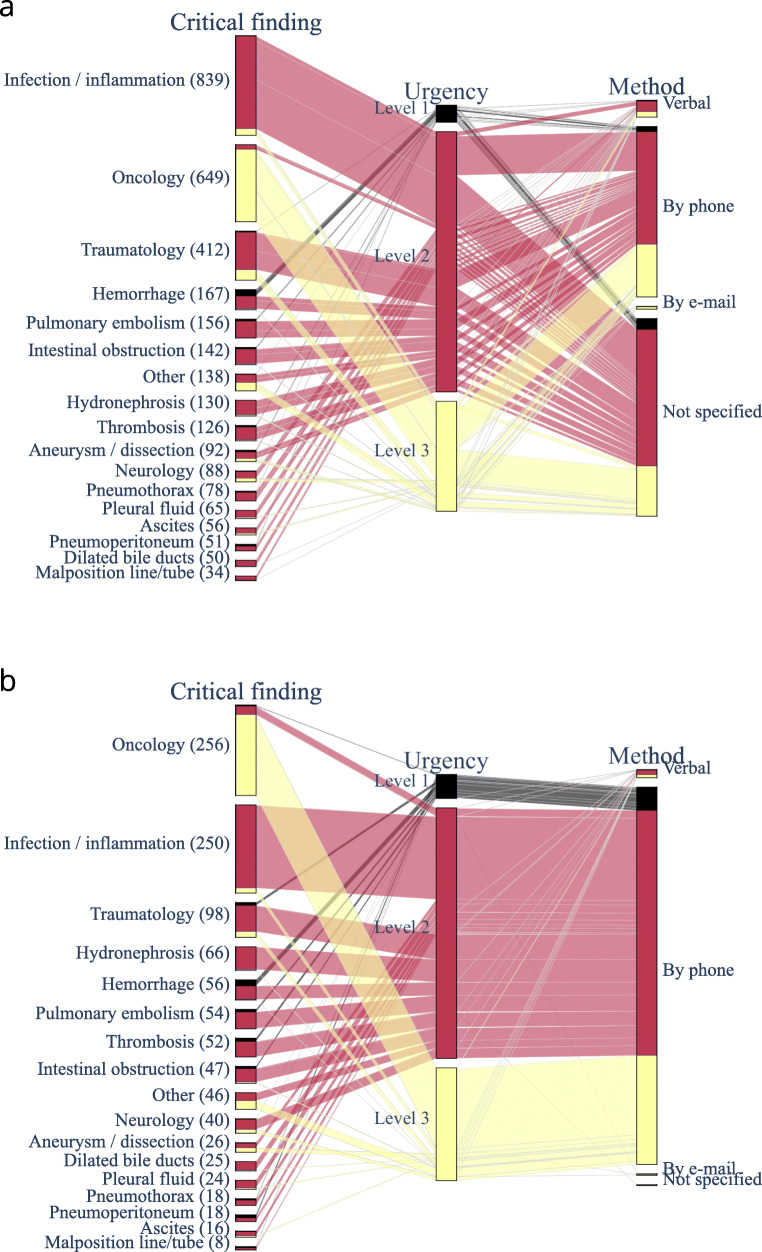
Information from all scored radiology reports without (a) and with (b) structured reporting usage is presented in parallel category diagrams. From left to right, the bars represent the critical finding category, the urgency level, and the method of communication as described in the report. The length of the bars represents the total number of reports, and the sections within a bar are proportional to the different categories. The colors represent the different urgency level categories

Figure 5 demonstrates an overview of the content and the process of the critical findings: the types of critical findings, the urgency level categories, and the method of communication. For both reports with and without structured reporting template, the two most often occurring categories are “infection/inflammation” and “oncology”, corresponding to a large part of urgency level 2 and level 3, respectively. For all three levels e-mail communication was nearly absent.

## Discussion

In this work, we present an implementation of contextual structured reporting to document the communication of critical findings in radiology reports. The template aides radiologists in documenting by the automated insertion of two context-dependent data elements (WHEN and HOW), and by guiding the radiologist to include the third required item (WHO). The voice command ensures the seamless integration in radiologist workflow. The rounded 100% score for all items indicates the technical feasibility of the system. The radiologists were required to dictate the name of the referring physician whom they contacted. The 99.7% adherence to this short-term post-implementation indicates that guiding of the radiologists to the specific insert-field was sufficient to facilitate them to perform the required action.

Clinical implementation was successful. A complete description of the communication of critical findings with all three required items (WHO, WHEN, HOW) was achieved in nearly 100% of the reports with template usage, compared to less than 5% of the reports without the template. This result was not only valid for a pre- versus post-implementation comparison (which is to be expected), but also in a post-implementation comparison between reports with and without a template. This difference illustrates the value of system-level interventions to improve quality.

The initial low compliance of template usage in the short-term post-implementation indicates that broad usage is not guaranteed. Technical feasibility does not immediately lead to broad usage. The high compliance in the long-term demonstrates that compliance was not restricted because of usability problems of the template itself. Increased awareness of guidelines and positive feedback probably contributed to the increased adherence.

Regarding the types of pathology and the urgency levels, the visual representation of fig. 5 demonstrates a similar pattern for reports with and without structured reporting template. This not unexpected because the radiologists were free to use the template for all types of pathology. We found no other comparative studies with a similar design.

For the process of communication, it is striking that even though non-direct communication, like e-mail, is allowed, radiologists preferred communication by phone. Probably the lack of an automated recording of an acknowledgement or receipt attributed to this. Closed-loop communication could otherwise only be obtained by manually checking the confirmation that the message was received and understood, or by implementing an alert notification system [17].

### Comparison with the literature

The DICOM Structured Reporting (SR) specification has been in use for over 20 years [18]. DICOM SR describes the technical specifications of integrating data elements in radiology reports, but implementation studies have been scarce in the years after introduction. Structured reporting studies usually emphasize clinical utility, with limited attention to technical details. Structured reports often consist of several template fields, with or without picklists, with a number of predetermined options for text within that field [19]. For our purpose, picklists would be less efficient compared to the automated insertion of data. A technical paper on Management of Radiology Report Templates (MRRT) illustrates the application of templates in the exchange of information in a clinical scenario of critical findings. Findings are coded to facilitate processing and automated notification of the referring physician [20]. Although the purpose and methodology differ from our study, we support the notion that technical solutions may improve efficiency and quality.

Our study is the first to describe an improved completeness of the communication of critical findings by implementing a structured reporting template. Our results are in line with previous studies, indicating that structured reporting implementation leads to more comprehensive radiology reports. Aase et al. demonstrate in a pre- and post-implementation study that template usage resulted in a higher percentage of required items in the description of incidental pulmonary nodules [4].

Another study on the documentation of the communication of critical findings (in neuroradiology only) also used a reporting template, but had a different purpose and methodology [21]. In this study a predefined list of critical findings was used and a random sample of selected reports was used. This allowed them to get insight into all reports with and without critical findings with the drawback of a relatively small sample of reports with critical findings (*n* = 195). Although our study lacks information about critical findings where the communication is not documented, this was not our study objective.

A broad range of guidelines is available for the management of different types of incidental and critical findings [22–27]. In addition to recommendations on managing incidental findings, several reports have been published on whether these recommendations improve the communication of critical findings [21, 28–32], and on the level of adherence to guidelines. For example, Clark et al. used free-text query software to identify studies investigating guideline adherence for two types of incidental findings (gallbladder polyps and thyroid nodules) [33]. As ours, this study also contributes to the evidence that quality and safety is improved by implementing guidelines and dedicated procedures, although the selected types of pathology and the methodology are different from our study.

We found no previous studies on the relationship between long-term usage and usability of structured reporting. Nevertheless, it is likely that these are associated, because in a study on usability measurement tools in eHealth, task completion was associated with usability [34].

In this study we analyzed the content of the critical findings by using manual annotation of radiology reports. Even though manual annotation can be regarded as gold standard, it is less suitable for continuous monitoring. Natural language processing is a promising method for automated monitoring and quality assurance of critical finding communication compliance [35].

### Limitations

The periods examined in our study have different durations. The pre-implementation and the short-term post-implementation periods are one year, compared to 2.5 months duration for long-term post-implementation. This difference is a limitation but did not have an impact on the results because of the sufficient large sample size compared with the difference between the groups. The short-term data demonstrated the significant change caused by the template. The long-term data proved usability and adherence.

Radiologists were offered the opportunity to use a structured reporting template, but they were not obliged to do so. This method could have introduced bias, in that radiologists that favour the use of structured reporting might beforehand be more likely to adhere to guidelines of critical findings than their colleagues who used free text.

We observed that post-implementation, not all radiologists used structured reporting, and radiologists who did use structured reporting did not do so for all of their reports with critical findings, at times opting to use free-text instead. Feedback during the evaluation period could have improved compliance with using the structured reporting template (Table 2).

Selection bias might have occurred as the communication of critical findings could also have been described in other, less-common words. It is unlikely that these cases impacted the results because this bias would have been the same in all three study groups.

Even though we based our data retrieval on a selection of Dutch words, our method of data extraction can be replicated in any language.

## Conclusion

Contextual structured reporting is feasible for required elements in radiology reporting and for automated insertion of context-dependent data.

Contextual structured reporting improves guideline adherence for communication of critical findings.
